# Technical Note: Design and commissioning of a water phantom for proton dosimetry in magnetic fields

**DOI:** 10.1002/mp.14605

**Published:** 2020-12-08

**Authors:** Hermann Fuchs, Fatima Padilla‐Cabal, Andreas Hummel, Dietmar Georg

**Affiliations:** ^1^ Division of Medical Radiation Physics Department of Radiation Oncology Medical University of Vienna Währinger Gürtel 18‐20 Wien 1090 Austria

**Keywords:** magnetic fields, MR‐guided proton therapy, particle therapy, proton therapy, water phantom

## Abstract

**Purpose:**

To design and commission a water phantom suitable for constrained environments and magnetic fields for magnetic resonance (MR)‐guided proton therapy.

**Methods:**

A phantom was designed, to enable precise, remote controlled detector positioning in water within the constrained environment of a magnet for MR‐guided proton therapy. The phantom consists of a PMMA enclosure whose outer dimensions of $81\times 40\times 12.5{\text{cm}}^{3}$ were chosen to optimize space usage inside the 13.5‐cm bore gap of the magnet. The moving mechanism is based on a low‐height H‐shaped non‐ferromagnetic belt drive, driven by stepper motors located outside of the magnetic field. The control system and the associated electronics were designed in house, with similar features as available in commercial water phantoms. Reproducibility as well as accuracy of the phantom positioning were tested using a high‐precision Leica AT 402 laser tracker. Laterally integrated depth dose curves and lateral beam profiles at three depths were acquired repeatedly for a 148.2 MeV proton beam in water.

**Results:**

The phantom was successfully operated with and without applied magnetic fields. For complex movements, a positioning uncertainty within 0.16 mm was found with an absolute accuracy typically below 0.3 mm. Laterally integrated depth dose curves agreed within 0.1 mm with data taken using a commercial water phantom. The lateral beam offset determined from beam profile measurements agreed well with data from Monte Carlo simulations.

**Conclusion:**

The phantom is optimally suited for detector positioning and dosimetric experiments within constrained environments in high magnetic fields.

## 1 INTRODUCTION

Proton and light‐ion beam therapy is an advanced form of cancer treatment. Their physical characteristics, especially their finite range, allow increasingly conformal dose distributions, reducing the total body dose.

In order to fully exploit the potential of this accurate dose delivery technique, image guidance is necessary to ensure the treated volume encompasses the target. Short and long term changes in the patient anatomy, for example, weight reduction, breathing, cardiovascular, or bowel movements, may have a non‐negligible impact on the delivered dose distribution. Improvements in image guidance would allow to reduce treatment margins, further improving the treatment efficacy.

Over the last years, the use of magnetic resonance (MR) guidance in radiation oncology is rapidly increasing. Compared to standard x‐ray‐based imaging solutions it offers superior soft tissue contrast with the additional advantage of zero imaging dose. Consequently, the combination of radiation therapy machines and MR guidance is an ongoing technological development. For photon‐based treatments, two vendors offer MR linacs, encompassing a 6MV linear accelerator and a MR system. Both hybrid MR linac systems started clinical operations recently.^1^, ^2^

In proton and light‐ion beam therapy, the development of a hybrid MR treatment system is still in its infancy. In contrast to photon beam therapy, the primary treatment beam itself is influenced by the magnetic field.^3^, ^4^, ^5^, ^6^ The impact of magnetic fields on the dose distribution, treatment delivery system as well as dosimetry were mostly studied textitin silico as only few facilities exist offering magnetic fields in a proton therapy research room.^7^, ^8^, ^9^, ^10^, ^11^, ^12^ In cooperation of the Medical University of Vienna and the MedAustron ion therapy center, a research magnet, capable of creating magnetic field strengths up to 1  T, was installed in an experimental particle therapy beam line.

Only very limited experimental dosimetry in magnetic fields have been performed so far.^9^ This is further complicated as there is no commercial equipment for proton dosimetry in the presence of magnetic fields available. Water is the preferred medium for precision dosimetry due to the easy standardization and established international standards (Ref. [^13^, ^14^]). Unfortunately, the space constraints of a research magnet imposes additional challenges and does not permit the use of previously designed phantoms.^15^ So far, no commercial platform allowing dosimetry in water with accurate detector positioning within the constraint volume and magnetic field of a research magnet, is available. This manuscripts describes the design and commissioning of such a dedicated, motorized water phantom for precision dosimetry in magnetic fields.

## 2 MATERIALS AND METHODS

### 2.1 Research magnet

**Fig 1:**
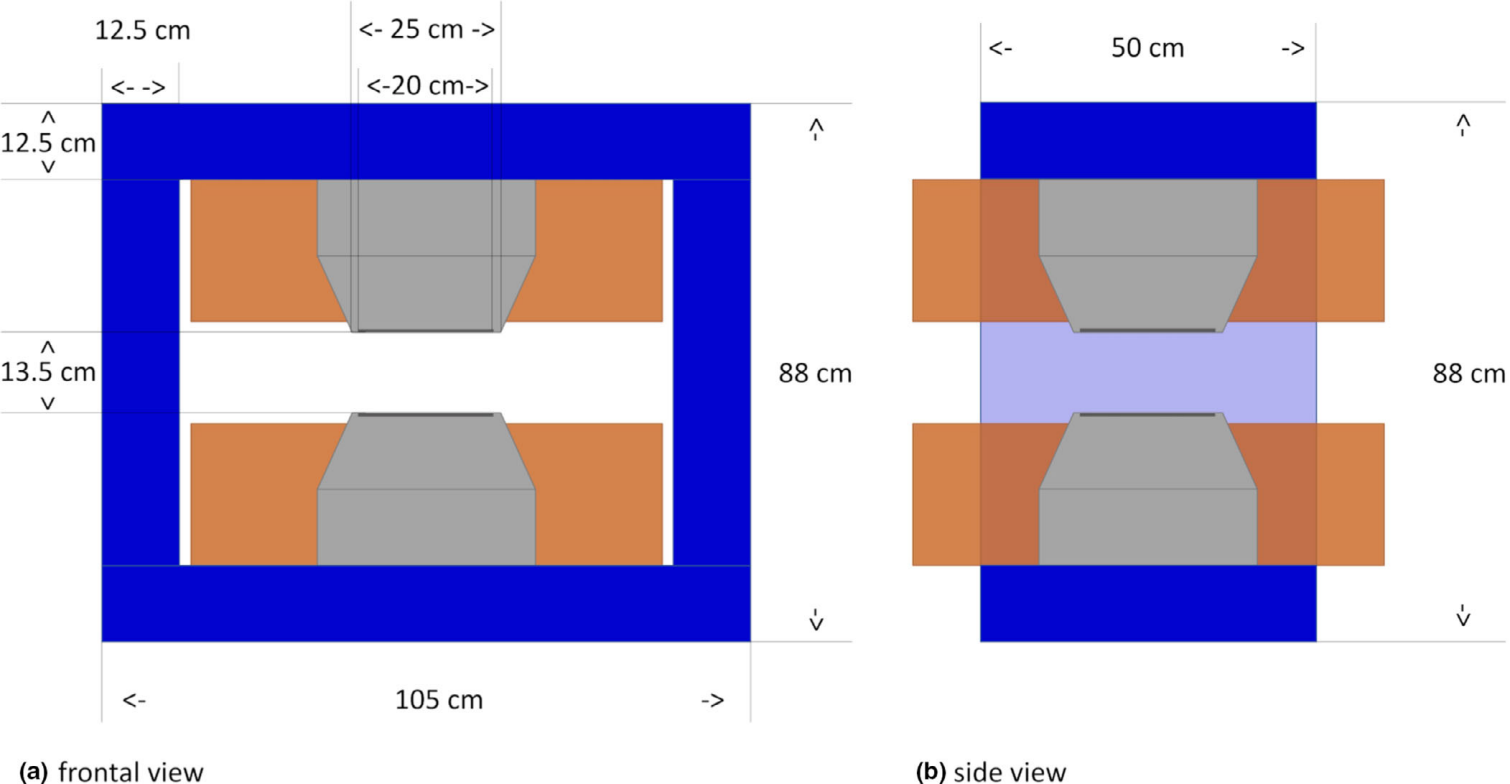
Illustration of the research magnet including dimensions. Magnet coils are indicated in brown, pole shoes in gray and magnet yokes in blue. Left hand side: frontal view. Right hand side: side view. [Color figure can be viewed at wileyonlinelibrary.com]

The research magnet, designed and constructed by Danfysik (Taastrup, Denmark), is a resistive, H‐shaped dipole magnet. It features an effective air gap of 13.5 cm between pole shoes, having a diameter of 25 cm (see Fig. 1). The magnet coils are water cooled, rated for a maximum current of 200 A, featuring a nominal field strength of 1 T at the center. Magnetic fringe fields fall off quickly, reaching ambient level at a distance of 50 cm from the center.

The magnet is mounted on a support system with heavy duty wheels, allowing easy positioning at the isocenter of the research beam line at MedAustron. Below the magnet, holding cabinets for the power supply system are located.

The total weight including the magnet, support, and power supply system is approximately 5 tons.

### 2.2 Water phantom

The MedAustron research rooms allows scanned field sizes of $20\times 20{\text{cm}}^{2}$ with a maximum range in water of 27 and 38 cm for proton and carbon ions, respectively. To avoid irradiating the magnet, field sizes are restricted to $20\times 11{\text{cm}}^{2}$ with the magnet in place.

The water phantom consists of 15‐mm thick PMMA plates glued together to form a container with outer dimensions of $81\times 40\times 12.5{\text{cm}}^{3}$. The phantom was designed to extend out of the back of the magnet in order to facilitate access to the mounted detectors without removal of the phantom. The front plate was equipped with a $25\times 9.5{\text{cm}}^{2}$ machine‐milled entrance window of 5mm thickness, centered alongside the central beam axis. The surface was milled and polished to an average roughness value of $R_{a}=0.8\mu m$. The water equivalent thickness of the entrance window was calculated to be 5.9 mm, using a scaling factor of 1.18 from previous experience. This thickness was later on confirmed using range measurements. Special care was taken to allow detector positioning immediately after the entrance window. In order to facilitate the alignment process, the frontside and backside were engraved with cross hairs.

The water phantom is located inside the magnet with the help of aluminum guide rails and adjustable end stops, ensuring a safe and stable positioning within the magnet. The phantom is filled after positioning to avoid water spillage due to phantom movements. Final alignment is performed with the help of a cross‐line laser system. The water is removed using a self‐priming pump operated by a battery‐powered power drill, before phantom removal.

### 2.3 Moving mechanism

Severe restrictions were imposed on the design and materials used due to the magnetic field strength, water filling as well as the limited headroom inside the magnet. Several design options were investigated until the optimal solution was found.

**Fig 2:**
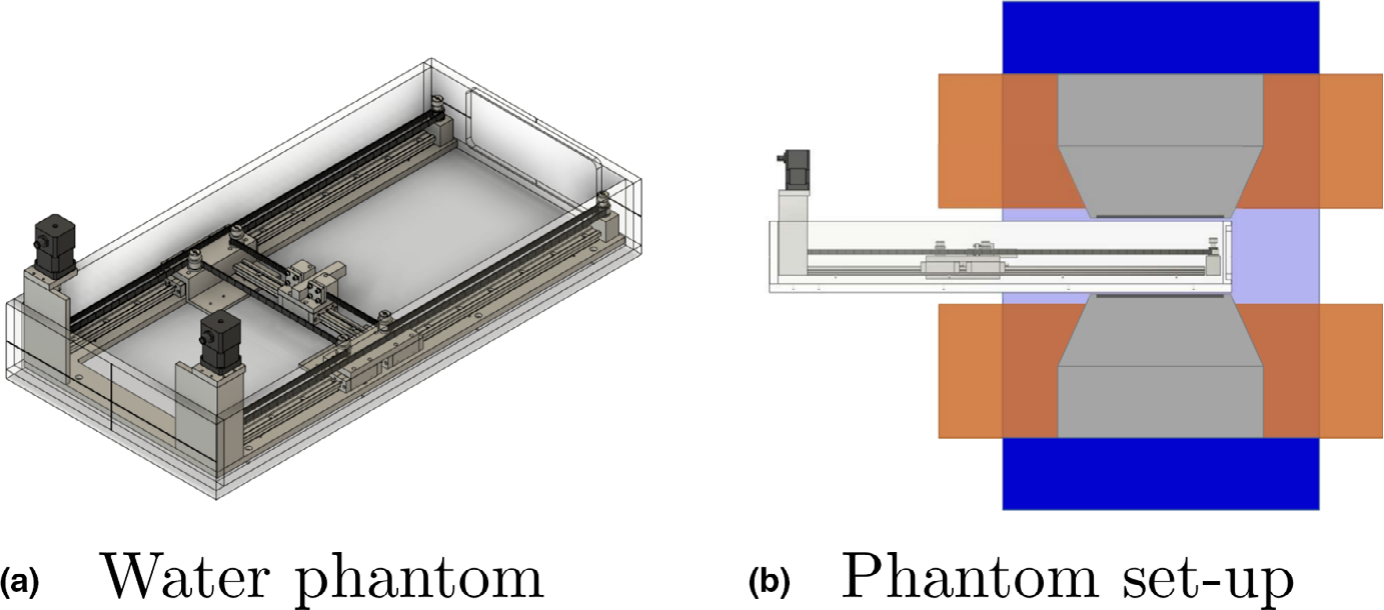
Left‐hand side [Fig. 2(a)] shows a sketch of the developed water phantom for high magnetic field environments. The entrance window is located on the right‐hand side of the phantom. The figure on the right hand side [Fig. 2(b)] illustrates the positioning of the phantom inside the magnet. [Color figure can be viewed at wileyonlinelibrary.com]

**Fig 3:**
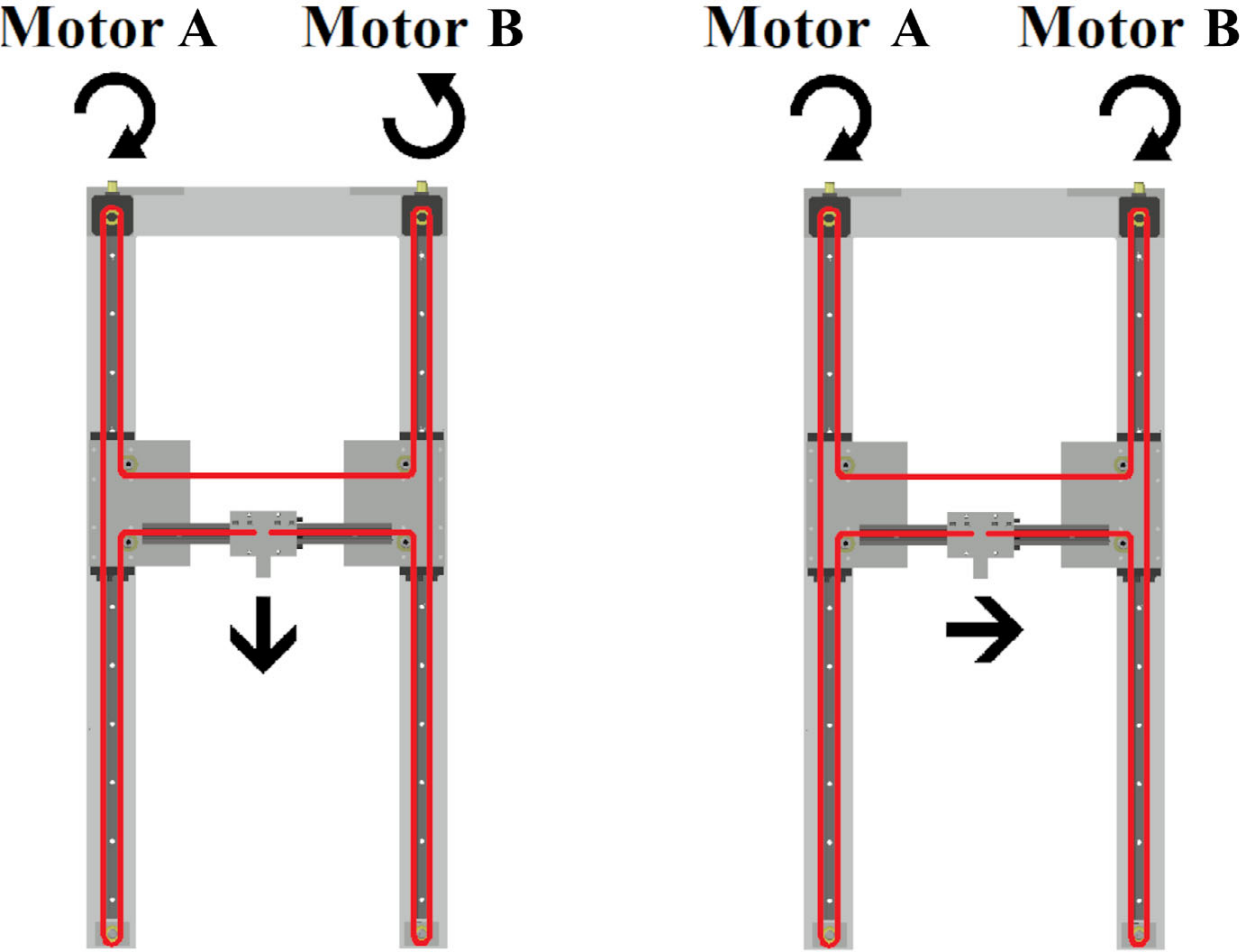
The H‐shaped 2D moving mechanism of the water phantom. The toothed belt is indicated with red lines. Depending on the rotation direction of the two stepper motors the detector holder moves longitudinal or lateral to the incident beam. [Color figure can be viewed at wileyonlinelibrary.com]

The moving mechanism allows computer controlled positioning in two dimensions: longitudinal and lateral to the incident beam direction. It is based on a H‐shaped belt drive system, which consists of a single timing belt, driven by two stepper motors. The stepper motors are mounted in the corners at the backside of the phantom, outside of the magnetic field (see Fig. 2). Consequently, standard NEMA17 stepper motors could be used. Two linear guides move alongside the longitudinal axis of the phantom, holding one lateral linear guide with the attached detector holder. The timing belt moves alongside the linear guides in an H‐shape, along the sides of the phantom and fixed to the detector holder. The detector holder is moved longitudinally to the beam incident axis if both motors operate in the opposite direction and laterally when both motors operate synchronously (see Fig. 3).

Drylin‐T guide rails (TS‐01) and carriage (TW‐01, Igus GmbH, Cologne, Germany) were used. Their total height of 24 mm makes optimal use of the restricted height available, allowing detector positioning in the center of the beam axis. In addition they are composed of aluminum alloys employing synthetic gliding elements instead of the usual steel ball bearings. A 9mm wide, 3M profile high‐torque drive teethed timing belt, consisting of neoprene with glass fiber cords, with nylon fabric covering of the running sides, was used to drive the detector holder carriage. Timing belt tension was set to 18N.

The whole moving mechanism is mounted on top of a 10‐mm thick aluminum base plate, so that it is self‐supported and to allow testing without the PMMA container. The base plate was designed to be U‐shaped to reduce the amount of metal in the incident beam path and fixed to the PMMA base plate.

Conversion of the movement distances to rotation angles for the stepper motors was performed using the following equations:

(1) $$\Delta \phi_{1}=\frac{‐\Delta x‐\Delta y}{r}$$

(2) $$\Delta \phi_{2}=\frac{‐\Delta x+\Delta y}{r}$$

where $\Delta \phi_{1}$ and $\Delta \phi_{2}$ correspond to the angle change of the stepper motors A and B, respectively, Δ*x* and Δ*y* to the change in lateral and longitudinal position, respectively, and *r* =  7.635  mm is the radius of the drive wheels. Due to mechanical tolerances, *r* was manually calibrated for optimal distance accuracy. Rotational speed of the motors was limited to 30 rotations per minute, corresponding to a maximum movement speed of about 2.3  cm/s.

### 2.4 Control system

The in house designed and built electronic control system enables remote control using standard ethernet cables and network infrastructure. An Arduino Uno R3 microcontroller board (Arduino SA, Chiasso, Switzerland), based on the Atmel ATmega328P microcontroller, was connected via two DRV8825 motor controllers to the stepper motors. The electronics used a standard 12 V power supply, while the stepper motors were equipped with a dedicated 320W 36V power supply. Ethernet connection was provided using an ARduino Ethernet shield 2, housing a W5500 Ethernet controller which was connected to the Atmel microcontroller via an SPI interface. Remote control was based on string commands, sent via the UDP protocol. For remote control, an in house developed python software was used. In addition, a local hand‐held featuring a LCD with a push‐button interface was created. This was connected to the control system by cable employing the $I^{2}C$ interface. The hand held controller supports most of the functions of the remote control and was designed to facilitate phantom setup. Functionality provided was similar to commercial water phantom control possibilities, such as zero point definition, movement limit restrictions and step size control.

### 2.5 Commissioning

The downside of the used non‐ferromagnetic linear rail system is a relatively large clearance for rotations. The belt system exhibits torsion on the drive carriages causing relatively large absolute positioning offsets, especially when changing the combined lateral and longitudinal movement direction. Initially, relative large hysteresis effects (up to 5 mm) were observed when changing movement direction. This effect was found to be negligible for the longitudinal direction alone. To mitigate these effects, a dedicated movement protocol was developed for high accuracy positioning. Lateral movements between measurement points are always performed in the positive direction. After a movement of the carriage in negative direction, a predefined starting movement of 5 mm was performed to allow a settling of the system. At the moment, this protocol is implemented manually, but at a later step may be included into the control software.

For commissioning, the phantom was set up at the isocenter of the irradiation room and aligned with the help of the in‐room laser system. A Leica AT 402 laser tracker was positioned in the irradiation room. A laser reflector was mounted on the detector holder of the phantom, to acquire absolute positions in the room coordinate system with uncertainties of −0.02, 0.04, and 0.03 mm for the three‐room coordinates, respectively. Laser tracker measurements were performed without the magnet as the magnet bulk interfered with the laser tracking system. Due to the manual orientation of the phantom based on in‐room lasers, a small residual alignment error was present. A three‐dimensional (3D) rotation matrix and offsets correction were applied to the measurements to compensate the alignment error. Measurements were performed with an empty phantom, as the laser reflector was not suitable for underwater operations.

Reproducibility of the phantom was tested by repeated movements to multiple specified positions, involving displacements along the longitudinal, lateral, or both axes. Position differences were evaluated by calculating the distance between nominal and measured position, taking into account all three room coordinates. Movement mechanism calibration was verified by commanding a 29 cm longitudinal movement and comparison to the absolute distance traveled. Overall positioning accuracy was evaluated using measurements alongside a rectangular grid covering an area of 15 × 45 cm with a spacing of 3 × 5 cm, for the lateral and longitudinal axis, respectively.

**Fig 4:**
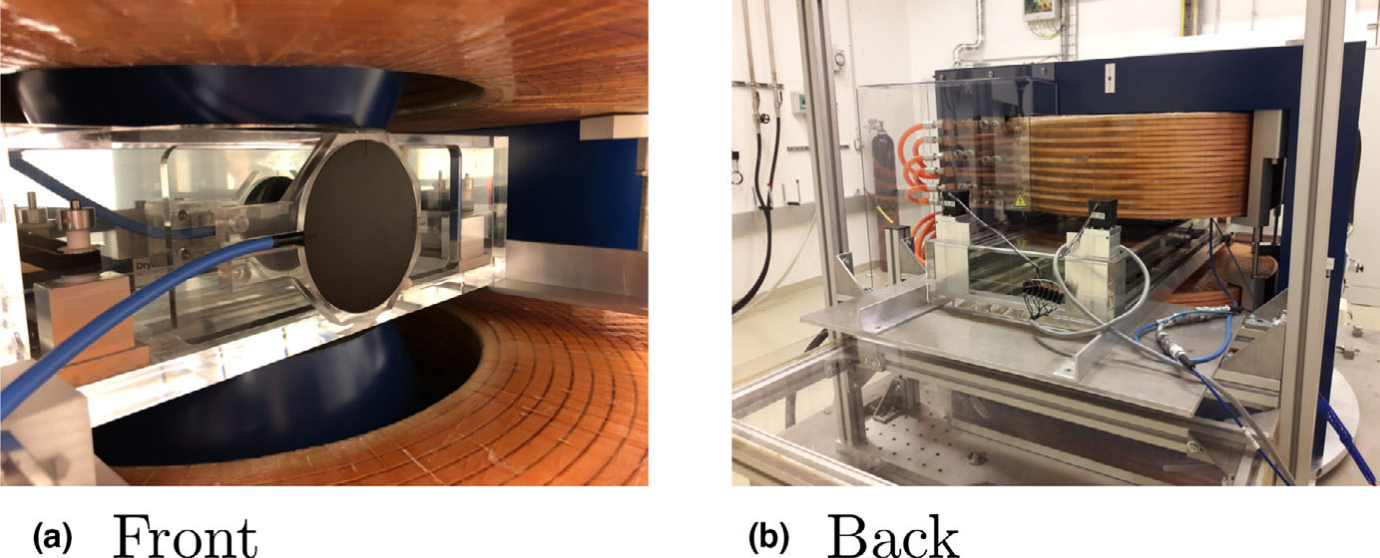
The left‐hand side [Fig. 4(a)] depicts the front side of the filled water phantom inside the magnet located in the isocenter of the research room, equipped with a small area Bragg Peak chamber (type 34073, PTW, Freiburg, Germany). Note the thin‐window Bragg‐peak chamber (type 34080, PTW, Freiburg, Germany) used for reference field measurement at the front of the phantom. The figure on the right‐hand side [Fig. 4(b)] depicts the back side of the water phantom inside the magnet. Note the black stepper motors located outside of the magnetic field. [Color figure can be viewed at wileyonlinelibrary.com]

Laterally integrated depth dose curves were repeatedly measured for a 148.2 MeV proton beam with and without an applied magnetic field of 1 T using a 48 mm plane parallel Bragg peak type 34073 (PTW, Freiburg, Germany) connected to an Unidos Webline (PTW, Freiburg, Germany). For reference, a thin window Bragg peak chamber type 34080 (PTW, Freiburg, Germany) was attached to the front of the water phantom and read out using a second Unidos Webline. Depth dose curves were compared to data measured during commissioning of the proton beam line using a 84 mm large area Bragg‐peak chamber (type 34070, PTW, Freiburg, Germany) and a commercial water phantom (MP3‐PL, PTW, Freiburg, Germany). A picture of the setup can be seen in Fig. 4. Particle range ($R_{80}$) was evaluated as the 80% dose maximum distal to the Bragg Peak. Bragg peak width (${\text{BPW}}_{80}$) was evaluated as the width between 80% of the dose proximal and distal of the Bragg Peak.

Using the same proton beam energy and magnetic field as for the longitudinal measurements, lateral beam profiles were measured in water at 20, 50, and 80% of the beam range. Measurements were performed employing a 0.016 ${\text{cm}}^{3}$ pin point thimble chamber (T31016, PTW, Freiburg, Germany) and the same reference chamber as before, read out by a Tandem XDR electrometer (PTW, Freiburg, Germany). Data were normalized to the profile maximum. Lateral beam profiles were compared to simulated profiles using the Monte Carlo toolkit GATE, which was validated previously by our group.^8^

## 3 RESULTS

The residual alignment error was determined and corrected. Rotational alignment errors were found to be −0.01, 0.04, and −0.03 degrees in Euler‐angles *ϕ*, *θ*, and *ψ*, respectively. The absolute positional offset was dependent on the zero point of the phantom and changed slightly between measurements due to re‐zeroing of the phantom. However, offsets ranged always between 0.1 and 0.5 mm for all three coordinate axes.

Excellent positioning reproducibility was found, with standard deviations of 0.14 and 0.09 mm for the separate lateral and longitudinal axes movements, covering translations between 15 and 29 cm, for both axes, respectively. Combined axes movements covering translations of 30 cm showed standard deviations of 0.16 mm.

**Fig 5:**
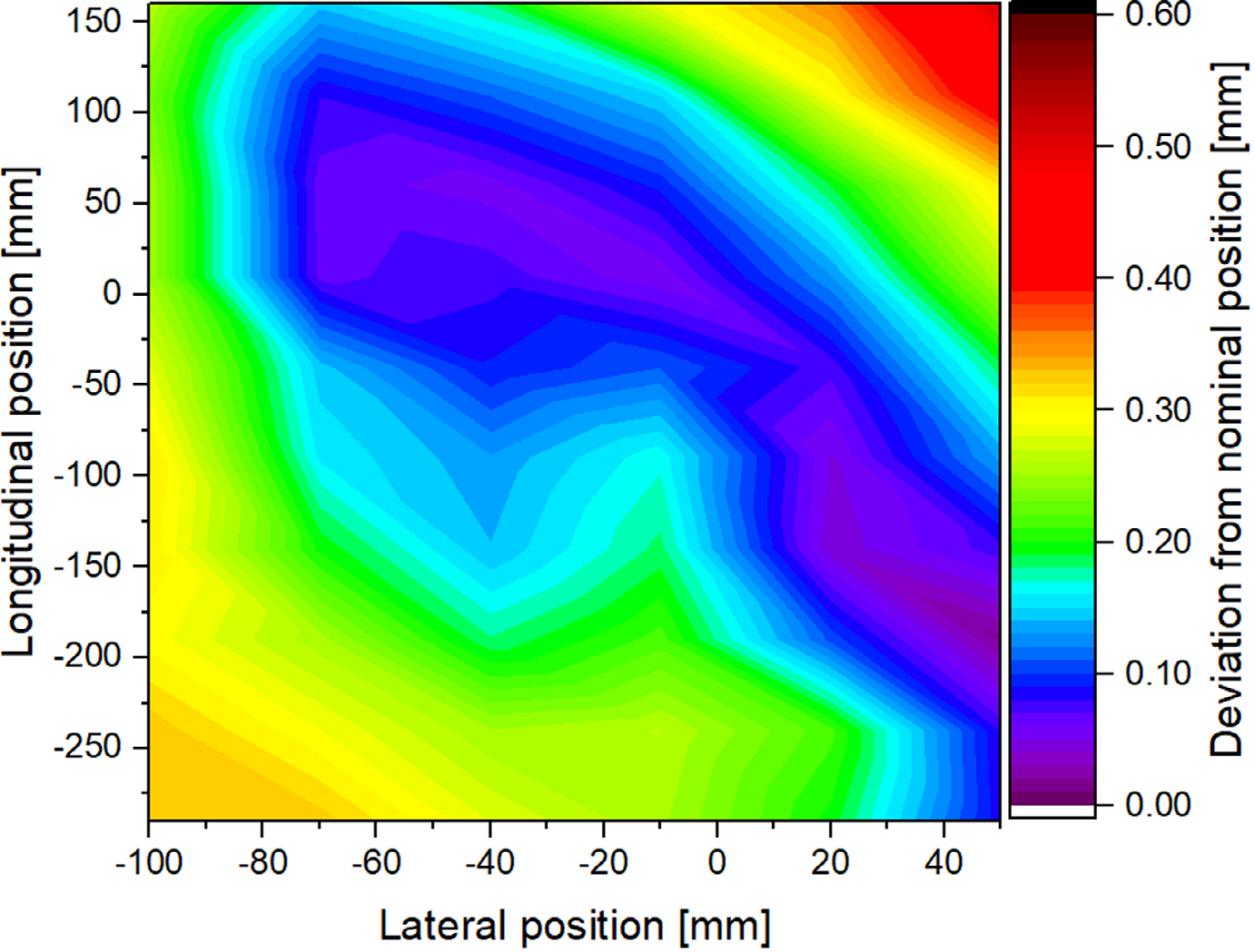
Absolute positioning deviations of the water phantom compared to the absolute room coordinate system. The zero point was chosen arbitrarily inside of the phantom. The center of the entrance window is located approximately at −25/−300 mm in lateral and longitudinal coordinates, respectively. [Color figure can be viewed at wileyonlinelibrary.com]

Calibration measurements showed a distance calibration error of −0.06%. As expected from the calibration offset, results of the absolute positioning uncertainty were found to depend on the distance to the arbitrarily chosen zero point of the phantom (see Fig. 5). Positions close to the phantom lateral borders showed higher deviations with a maximum deviation of 0.5 mm, and average absolute deviations of 0.2 mm.

**Fig 6:**
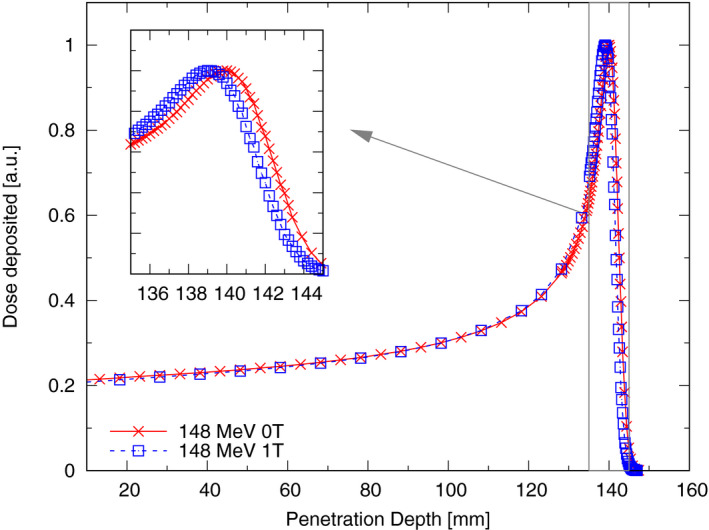
Laterally integrated depth dose profile of a 148.2 MeV proton beam in water with and without an applied magnetic field of 1 T, measured using a small diameter Bragg peak chamber (T34073, PTW, Freiburg, Germany). [Color figure can be viewed at wileyonlinelibrary.com]

Laterally integrated depth dose profiles agreed within 0.2 mm with the expected values (see Fig. 6 and Table I). For the 148.2 MeV beam before the Bragg‐peak values were slightly lower compared to the reference measurements due to the employed smaller chamber diameter. No difference in accuracy was found due to the additional drag from large detectors.

**Table I:** Range and Bragg peak width of a 148.2 MeV proton beam in water, determined using the in‐house developed water phantom (MR phantom) with and without magnetic fields, a commercial water phantom (reference), and Monte Carlo (MC) simulations.

Energy (MeV)	Measurement	Field strength (T)	$R_{80}$ (mm)	$BPW_{80}$ (mm)
148.2	MR phantom	0	150.2	4.1
		1	149.4	4.1
	Monte Carlo	0	150.2	4.2
		1	149.5	4.2
	Reference	0	150.2	4.2

**Fig 7:**
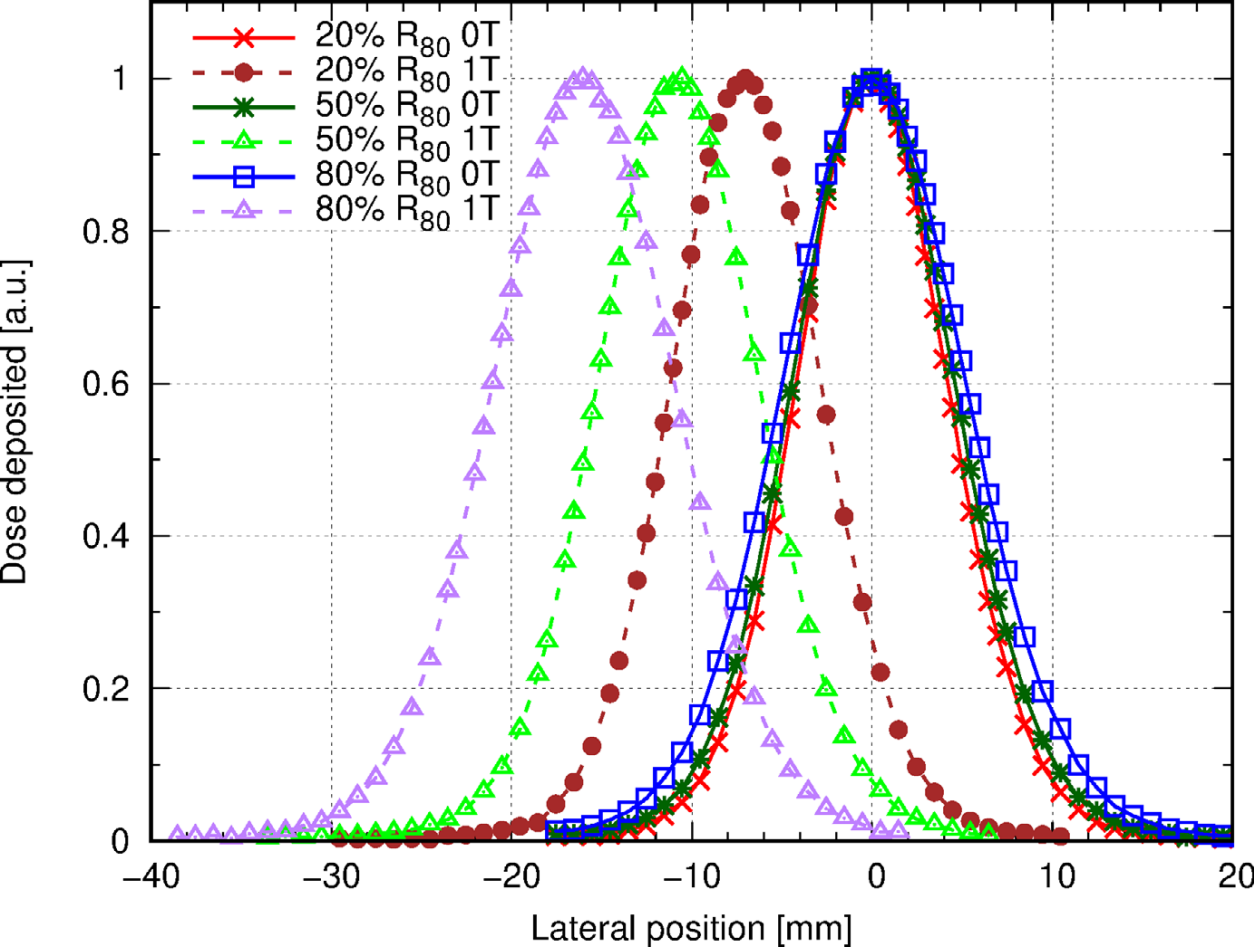
Laterally dose profile of a 148.2 MeV proton beam in water, measured at 20, 50, and 80% of the beam range using a 0.016 ${\text{cm}}^{3}$ pin point thimble chamber (T31016, PTW, Freiburg, Germany) with and without an applied magnetic field of 1 T. Data were normalized to the profile maximum. [Color figure can be viewed at wileyonlinelibrary.com]

Lateral dose profiles of a 148.2 MeV proton beam showed beam broadening and, for the magnetic field cases, as expected the deflection due to the magnetic field (see Fig. 7). A comparison of the lateral offsets with data from in‐house calculated Monte Carlo simulations can be found in Table II. Differences in beam width can be explained by averaging effects over the chamber volume. Differences between iterations were found to be less than 0.1 mm.

**Table II:** Comparison of experimental and Monte Carlo (MC) simulated lateral profiles of a 148.2 MeV proton beam in water at various positions with and without an applied magnetic field of 1 T.

Field strength (T)	Profile position	Beam center (mm)		Beam width (mm)	
		MC	Measured	MC	Measured
0	20% of $R_{80}$	0.0	0.0	4.0	4.2
	50% of $R_{80}$	0.0	0.0	4.3	4.5
	80% of $R_{80}$	0.0	0.0	4.9	5.1
1	20% of $R_{80}$	−7.0	−7.0	3.9	4.2
	50% of $R_{80}$	−11.4	−10.8	4.2	4.5
	80% of $R_{80}$	−16.3	−16.0	4.9	5.1

## 4 DISCUSSION

So far, little data are available for proton dosimetry in the presence of magnetic fields. However, it was already shown recently,^16^ detector positioning uncertainty may have a large impact on the overall dosimetric uncertainty. Especially when measuring with small fields, dosimetric errors of up to 20% per mm displacement uncertainty were reported. Single pencil beams in proton therapy, often measured for commissioning and used for precision measurements are in a similar size range. Consequently, special care was taken to ensure high precision and accuracy, as the absolute chamber position as well as the reproducibility may have a significant impact.

Measured and simulated longitudinal and lateral beam profiles agreed very well. For the lateral beam profile offset, a slightly larger deviation was seen for the Monte Carlo values, which may be due to inaccuracies while positioning within the magnet and consequently slightly different magnetic fringe fields.

So far, no data on the response of ion chambers inside magnetic fields for proton beam therapy have been published. Due to the different physical processes and energy spectra of secondary particles, it is not possible to directly relate correction factors determined using photon irradiation. However, as the magnetic field strength during our measurements did not change, potential correction factors should also be constant. Consequently, only relative measurements were performed.

Bridging the gap from such scientific requirements to engineering reality proved challenging. In many areas, new design ideas and technologies had to be introduced to reach and verify the required system performance. The biggest design obstacle was the combination of constraint space, water and magnetic field. Most available positioning systems were either too large, not water proof or contained ferromagnetic materials. Conventional linear axes were either too large, or would have required the placement of a motor inside the magnetic field and in water. Although some ultrasound motors suitable for magnetic fields are available and used in commercial MR phantoms, these were not suitable for submersed operation.

The use of sensors to increase absolute phantom positioning was not suitable. Due to the challenging environmental conditions, space limitations and accuracy requirements, potential optical commercial sensor systems were found to be far outside an affordable price range. A future upgrade could include the introduction of laser fiber optics‐based position sensors; however, so far such sensors proved too expensive and cumbersome to handle in the required accuracy range. An alternative improvement might be the definition of a reference position employing an end switch which could be checked automatically. However, the developed movement protocol was able to considerably reduce positioning uncertainty.

To further improve reproducibility, the belt tension of the movement system was increased. Initial calibration was performed with an adequate, but slightly lower belt tension. Although the belt tension increase lead to a better overall performance of the system, it may have slightly compressed the belt around the motor pulleys, leading to the observed distance deviations. We have so far not observed a change in belt tension (over a period of 6 months). Nevertheless, for long‐term use. checks on belt tension should be part of regular quality control. However, in most experiments, the zero point will be chosen close to the positions of interest, consequently reducing the influence of the calibration uncertainty below the reproducibility of the system.

Performing position measurements in the submillimeter scale over large areas proved challenging with conventional tools. The use of the laser tracker system allowed precise measurements, but was limited to the coordinate system of the research room. Consequently, alignment errors of the water phantom within the research room had to be corrected, potentially artificially increasing the uncertainties of the system.

The chosen belt system does not require a synchronized operation of the motors, as the end position is determined by the overall rotation angles of the motors. However, although the end position is the same, as the motors are not synchronized, the path to the final position may vary a slightly. Consequently, small additional margins have to be added to the movement limits or special care has to be taken with the potential carriage paths.

The phantom dimensions were chosen to be as big as feasible, allowing access to the mounted detectors without having to remove the phantom from the magnet. Wall thicknesses were chosen with a considerable safety margin to keep the phantom as sturdy as possible and to prevent deformations by the water filling. The current movement system was mounted on aluminum base plate, to be self‐supporting and to allow testing outside of the PMMA container. This has the additional benefit of potentially using the positioning system without the PMMA container for other experiments at a later stage.

Currently, standard stepper motors are employed, as this part of the phantom was not planned to be exposed to a magnetic field. A later upgrade to magnetic field‐resistant ultrasound‐based motors is possible, allowing the use of the phantom in even larger magnetic field setups.

## 5 CONCLUSION

The in‐house developed water phantom showed comparable performance to commercial water phantoms. With an absolute positioning repeatability of 0.16 mm and accuracy typically in the order of 0.3 mm, the phantom, built from non‐ferromagnetic components including the detector positioning system, is optimally suited for dosimetry and experiments in high magnetic fields.
